# Invasive Infection With *emm3*/ST15 *Streptococcus pyogenes*: The First Case Report From China and Complete Genome Analysis

**DOI:** 10.3389/fmed.2022.861087

**Published:** 2022-05-09

**Authors:** Xinli Mu, Yanfei Wang, Lu Sun, Shanshan Zhao, Xi Jin, Junli Zhang, Yunsong Yu, Xueqing Wu

**Affiliations:** ^1^Department of Infectious Diseases, Sir Run Run Shaw Hospital, Regional Medical Center for National Institute of Respiratory Diseases, Key Laboratory of Microbial Technology and Bioinformatics of Zhejiang Province, Hangzhou, China; ^2^Department of Clinical Laboratory, Shangyu People’s Hospital, Shaoxing, China; ^3^Centre of Laboratory Medicine, Zhejiang Provincial People’s Hospital, People’s Hospital of Hangzhou Medical College, Hangzhou, China

**Keywords:** *Streptococcus pyogenes*, M type, *emm3*/ST15, invasive infection, whole-genome sequencing (WGS)

## Abstract

*Streptococcus pyogenes* (GAS) may cause severe invasive disease with a high fatality rate, especially M3-type strains, which are less common in China. Here, we report the first *emm3*/ST15 invasive GAS infection case in China. The patient was diagnosed with severe skin and soft tissue infection (SSTI) and septicaemia caused by one GAS strain. Antibiotic susceptibility tests showed that the isolate was susceptible to all tested drugs. Antimicrobial therapy was then applied, and the patient fully recovered and was discharged from the hospital on Day 43. Whole-genome sequencing was carried out using the Illumina and Oxford Nanopore platforms and revealed this to be the first *emm3*/ST15-type GAS invasive infection in China. The closely related *emm3*/ST15-type GAS strains are MGAS315 from the United States and M3-b from Japan. Our finding is a warning that we should pay attention to invasive M3-type GAS infections in China and indicates the global spread of the highly virulent emm3/ST15 GAS strain.

## Introduction

*Streptococcus pyogenes* (Group A Streptococcus, GAS) is a common Gram-positive pathogenic bacterium that may induce various diseases, including minor ones, such as pharyngitis and scarlet fever, and serious ones, such as pneumonia and toxic shock syndrome (1). Severe GAS infections are less common but present a high fatality rate and have been reported worldwide (2–4). For example, the symptoms of GAS pharyngitis include fever, throat pain, and chills, while streptococcal toxic shock syndrome patients would develop multiorgan failure within a short time, leading to death (5, 6). It was reported a combined antibiotic therapy of penicillin and clindamycin would improve the outcomes of severe GAS infections (7).

The transmission of GAS would be through direct person-to-person *via* the inhalation of respiratory droplets, or through direct contact with contaminated objects (8). The increment in the incidence of GAS invasive infections has been associated with particular clones and varies by time and region, which may reflect a population’s susceptibility to specific strains (9, 10). GAS strains can be serologically separated into M protein serotypes based on a surface protein encoded by the *emm* gene (1). In clinical epidemiological studies, M1 and 3 are the most common GAS serotypes of invasive and toxic streptococcal diseases. Among the different sequence types of M3 strains, *emm3*/ST15-induced toxic shock syndrome and other invasive diseases have often been reported in Japan, United Kingdom, and United States (11).

In the past, we considered that M3 GAS infections were rare in China, but a recent large-scale epidemiological study indicated that M3 GAS scarlet fever has increased substantially in China since 2018 (12) and became one of the prevalent *emm* types that induced scarlet fever in 2019 (13). Here, we report the first case of a severe GAS infection caused by the *emm3*/ST15 GAS strain SHZ-1 in China. The complete genome sequence of this strain was also analyzed in the current study.

## Methods

### Bacterial Isolation and Antimicrobial Susceptibility Testing

Wound secretion specimens were submitted to the clinical laboratory of Zhejiang local hospital for slide microscopy and bacterial culture using 5% sheep blood agar plates. A GAS strain was obtained and identified by matrix-assisted laser desorption ionization-time of flight mass spectrometry (Bruker Daltonics, Bremen, Germany) (14). Antimicrobial susceptibility testing (AST) of levofloxacin, chloramphenicol, clindamycin, ceftriaxone, erythromycin, penicillin, tetracycline, and vancomycin against the identified GAS strain, named SHZ-1, was performed using a disk diffusion method, and the results were interpreted according to the Clinical and Laboratory Standards Institute (15).

### Whole-Genome Sequencing and Analysis

Genomic DNA of strain SHZ-1 (single colony) was prepared using a DNA mini kit (Qiagen, Valencia, CA, United States) and submitted to next-generation sequencing (NGS) and long-read sequencing using the Illumina HiSeq2000TM (Illumina Inc., San Diego, CA, United States) and Oxford Nanopore MinION platforms (Oxford Nanopore Technologies, Oxford, United Kingdom), respectively. The Illumina sequencing generated 9.1M reads with an average of ∼150 bp per read and 200 X coverage. The nanopore sequencing of this strain generated 139 contigs with an N50 of 1329619 bp and a GC content of 39.22%. Both short and long reads were used for *de novo* assembly *via* Unicycler version 1.0 (15) in a hybrid assembly model with default parameters. The genome was then used as input for *in silico* multilocus sequence typing (MLST) *via* BLAST against the PubMLST database (16), *emm* typing against the CDC emm type and subtype database tsemm (17). The annotation of SHZ-1 was performed *via* Prokka against *S. pyogenes* database (18). The annotated genome was then used for virulence factor detection against the VFDB database (19). Both SNP and cgMLST strategies were performed in the BacWGSTdb server (20) for bacterial source tracking. An *ad hoc* cgMLST scheme for *S. pyogenes* (1,170 target genes) was designed to characterize the gene-by-gene allelic profile of *S. pyogenes* strains. The identified *S. pyogenes* strain SHZ-1 was used as the reference genome in this analysis. SNP distance matrices were calculated using snp-dists 0.6.3 (21).

## Results

### Case Presentation

**FIGURE 1:**
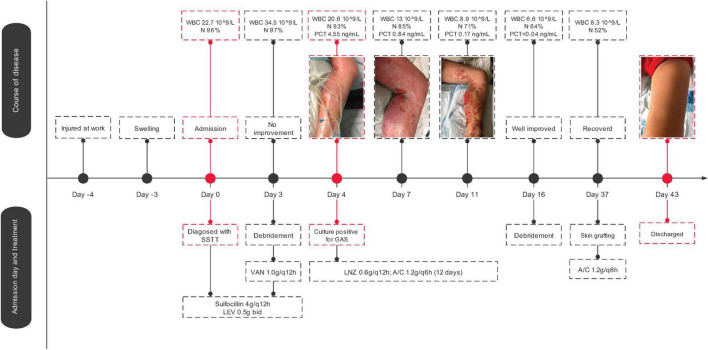
*Streptococcus pyogenes* strain SHZ-1 infection history and genomic epidemiological analysis. The medical history of SHZ-1 infected patient. Abbreviations in the figure: SSTI, skin and soft tissue infection; LEV, Levofloxacin; VAN, vancomycin; LNZ, linezolid; WBC, white blood cell; N, neutrophils; PCT, procalcitonin; A/C, amoxicillin/clavulanate.

A 37-year-old man injured his left foot at work 4 days prior to admission. He immediately felt pain, but it was tolerable and he had no open wounds; hence, he ignored the symptoms. The injured foot developed skin redness, swelling, and obvious tenderness 1 day later. The patient developed a conscious fever accompanied by chills, dizziness, abdominal pain, and distension. The symptoms worsened after another 2 days, and the patient was then admitted to the local hospital, Zhejiang, China. His lab results showed leukocyte counts of 22.3 × 10^9^/L with 92.3% neutrophils, and an X-ray showed no fracture. Based on this, the diagnoses of severe skin and soft tissue infection (SSTI) was made. Three days of sulbenicillin (4 g/q12 h) and levofloxacin (0.5 g bid) treatment showed no improvement. After debridement, postoperative anti-infection treatment with vancomycin (1.0/q8 h) was applied. Leukocyte counts were still high with elevated levels of procalcitonin, and the patient was diagnosed with septicemia. X-ray confirmed no migrating lung lesions. On the fourth day of admission, a specimen culture was confirmed to be positive for GAS. AST showed that the bacterial isolate was susceptible to all tested drugs (Supplementary Table 2). Antimicrobial therapy was then applied with linezolid (0.6 g q12 h) and amoxicillin/clavulanate potassium (1.2 g q6 h). Declines in all inflammatory indices were then observed. Thereafter, the patient improved from GAS infection after 11 days of treatment and was transferred to debridement, suture, and skin grafting. The patient was treated with amoxicillin/clavulanate potassium (1.2 g q8 h) for another 6 days since Day 37 due to GAS culture positivity. The patient fully recovered and was discharged from the hospital on Day 43 (Figure 1).

### Whole-Genome Analysis

**FIGURE 2:**
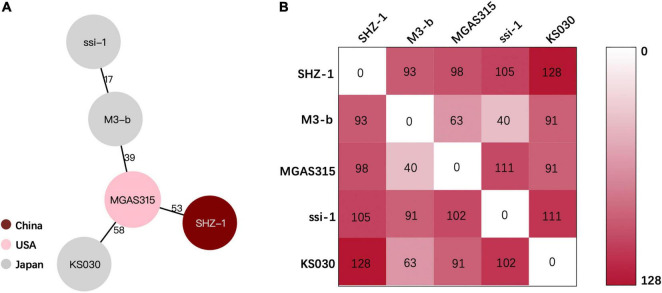
Phylogenetic relationship between SHZ-1 and the closely related *Streptococcus pyogenes* strains. **(A)** The lines connecting the circles indicate the clonal relationship between different isolates and the digital numbers on the lines illustrate the number of allelic differences. **(B)** The single-nucleotide polymorphisms (SNPs) numbers between each *S. pyogenes* strain. The gradient of purple intensity illustrates the quantity of SNPs.

Hypervirulent invasive GAS infections are rare in China. To better understand the pathogen in the current case, the strain was sent for whole-genome sequencing. The complete chromosome data of SHZ-1 were submitted to NCBI (Genbank No.CP072523.1). After the hybrid assembly, we obtained a 1819973 bp complete circular genome of GAS strain SHZ-1 with a GC content of 38.6%. We identified SHZ-1 as an *emm3*/ST15-type GAS strain *via emm* typing and MLST analysis (Supplementary Figure). Then we downloaded the complete genome of *emm3*/ST15 GAS strains that were isolated from different regions. Using BacWGSTdb to determine the clonal relationship among these isolates according to the pairwise comparison of the cgMLST alleles or SNP differences. In the end, the phylogenetic relatedness showed that the closest strain to SHZ-1 was MGAS315 from the United States, which has 53 core gene differences (Figure 2A). The SNP calling results indicated that the smallest SNP number of 93 was determined between strains SHZ-1 and M3-b from Japan (Figure 2B). Virulence factor detection against VFDB showed that SHZ-1 had all the virulence factors found in the other two *emm3*/ST15 strains (MGA315 and M3-b) except the streptococcal superantigen SSA-encoding gene *ssa* (Supplementary Table 2).

## Discussion

*Streptococcus pyogenes* serotype M3 strain-induced invasive infections have been widely reported worldwide, although they have not been common in recent years in China. We report the first case of the *emm3*/ST15 GAS strain causing severe invasive infection in China.

Recent large-scale epidemiological studies have reported an M-type shift of scarlet fever GAS isolates in China, which clearly showed that M3 GAS has become one of the most prevalent strains in Beijing, China, since 2018 (1, 2). Our reported severe infection case indicates the emergence of widespread M3 strains in China, which belong to the most virulent strain *emm3*/ST15. Whole-genome analysis of SHZ-1 showed that the closest strain was MGAS315 from United States (22) and then M3-b from Japan (23). Both strains are invasive isolates from patients with streptococcal toxic shock syndrome in the late 1980s and 1994, respectively. Later, whole-genome analysis of these two and other *emm3*/ST15 strains showed that their association with severe infections was due to their distinct arrays of virulence factors, for example, *speA*-encoded streptococcal pyrogenic exotoxin A and *ssa*-encoded streptococcal superantigen A (22). The latter is a phage-associated streptococcal superantigen that is missing in our reported strain SHZ-1 but is encoded in both the MGAS315 and M2-b genomes. *ssa* has been detected in toxic shock syndrome-related M3 isolates (24), but only 25% of *emm3*-type GAS isolates are from Chinese patients (13). In our reported case, strain SHZ-1 that lacked the *ssa* gene still presented a strong pathogenesis in the host. We believe that genetically related *emm*/ST15 GAS strains may have geographic characteristics for their virulence, and a large-scale molecular epidemiology study of invasive GAS infection in China is needed.

In conclusion, we report the first *emm3*/ST15 invasive GAS infection in China. The complete genome analysis of pathogenic GAS strain SHZ-1 indicates the emergence of the global spread of highly virulent *emm3*/ST15-type GAS. This is a warning that attention should be paid to the epidemiology of M3-type GAS in China to prevent severe invasive GAS infections.

## Ethics Statement

Written informed consent was obtained from the patient for the publication of any potentially identifiable images or data included in this article.

## Author Contributions

XM, JZ, and LS collected the clinical data. YW and SZ did the laboratory work. XW and YY designed the study and wrote the manuscript. All authors contributed to the article and approved the submitted version.

## Conflict of Interest

The authors declare that the research was conducted in the absence of any commercial or financial relationships that could be construed as a potential conflict of interest.

## Publisher’s Note

All claims expressed in this article are solely those of the authors and do not necessarily represent those of their affiliated organizations, or those of the publisher, the editors and the reviewers. Any product that may be evaluated in this article, or claim that may be made by its manufacturer, is not guaranteed or endorsed by the publisher.

## References

[1] Cunningham MW. Pathogenesis of group A streptococcal infections. *Clin Microbiol Rev.* (2000) 13:470–511. 10.1128/CMR.13.3.470 10885988 PMC88944

[2] Wong CJ Stevens DL. Serious group a streptococcal infections. *Med Clin North Am.* (2013) 97:721–36. 10.1016/j.mcna.2013.03.003 23809722

[3] Blagden S Watts V Verlander NQ Pegorie M. Invasive group A streptococcal infections in North West England: epidemiology, risk factors and fatal infection. *Public Health.* (2020) 186:63–70. 10.1016/j.puhe.2020.06.007 32784097

[4] Leung TN Hon KL Leung AK. Group A *Streptococcus* disease in Hong Kong children: an overview. *Hong Kong Med J.* (2018) 24:593–601. 10.12809/hkmj187275 30416105

[5] Mustafa Z Ghaffari M. Diagnostic methods, clinical guidelines, and antibiotic treatment for group a streptococcal pharyngitis: a narrative review. *Front Cell Infect Microbiol.* (2020) 10:563627. 10.3389/fcimb.2020.563627 33178623 PMC7593338

[6] Belkassa K Khelifa M Batonneau-Gener I Marouf-Khelifa K Khelifa A. Understanding of the mechanism of crystal violet adsorption on modified halloysite: hydrophobicity, performance, and interaction. *J Hazard Mater.* (2021) 415:125656. 10.1016/j.jhazmat.2021.125656 33756196

[7] Babiker A Li X Lai YL Strich JR Warner S Sarzynski S Effectiveness of adjunctive clindamycin in beta-lactam antibiotic-treated patients with invasive beta-haemolytic streptococcal infections in US hospitals: a retrospective multicentre cohort study. *Lancet Infect Dis.* (2021) 21:697–710. 10.1016/S1473-309930523-5 33333013 PMC8084921

[8] Kemble SK Westbrook A Lynfield R Bogard A Koktavy N Gall K Foodborne outbreak of group a *Streptococcus* pharyngitis associated with a high school dance team banquet–Minnesota, 2012. *Clin Infect Dis.* (2013) 57:648–54. 10.1093/cid/cit359 23868521

[9] Luca-Harari B Darenberg J Neal S Siljander T Strakova L Tanna A Clinical and microbiological characteristics of severe *Streptococcus pyogenes* disease in Europe. *J Clin Microbiol.* (2009) 47:1155–65. 10.1128/JCM.02155-08 19158266 PMC2668334

[10] O’Brien KL Beall B Barrett NL Cieslak PR Reingold A Farley MM Epidemiology of invasive group a *Streptococcus* disease in the United States, 1995-1999. *Clin Infect Dis.* (2002) 35:268–76. 10.1086/341409 12115092

[11] Sekizuka T Nai E Yoshida T Endo S Hamajima E Akiyama S Streptococcal toxic shock syndrome caused by the dissemination of an invasive emm3/ST15 strain of *Streptococcus pyogenes*. *BMC Infect Dis.* (2017) 17:774. 10.1186/s12879-017-2870-2 29254479 PMC5735678

[12] You Y Peng X Yang P Wang Q Zhang J. 8-year M type surveillance of *Streptococcus pyogenes* in China. *Lancet Infect Dis.* (2020) 20:24–5. 10.1016/S1473-309930694-2 31876494

[13] Li H Zhou L Zhao Y Ma L Xu J Liu Y Epidemiological analysis of group A *Streptococcus* infections in a hospital in Beijing. *China. Eur J Clin Microbiol Infect Dis.* (2020) 39:2361–71. 10.1007/s10096-020-03987-5 32676802

[14] Croxatto A Prod’hom G Greub G. Applications of MALDI-TOF mass spectrometry in clinical diagnostic microbiology. *FEMS Microbiol Rev.* (2012) 36:380–407. 10.1111/j.1574-6976.2011.00298.x 22092265

[15] Wayne PA CLSI. *Performance Standards for Antimicrobial Susceptibility Testing.* 28th ed. Wayne, PA: Clinical and Laboratory Standards Institute (2018).

[16] Enright MC Spratt BG Kalia A Cross JH Bessen DE. Multilocus sequence typing of *Streptococcus pyogenes* and the relationships between emm type and clone. *Infect Immun.* (2001) 69:2416–27. 10.1128/IAI.69.4.2416-2427.2001 11254602 PMC98174

[17] CDC. *Streptococcus* Laboratory: Centers for Disease Control and Prevention. Atlanta, GA: Centers for Disease Control and Prevention (1949).

[18] Seemann T. Prokka: rapid prokaryotic genome annotation. *Bioinformatics.* (2014) 30:2068–9. 10.1093/bioinformatics/btu153 24642063

[19] Chen L Yang J Yu J Yao Z Sun L Shen Y VFDB: a reference database for bacterial virulence factors. *Nucleic Acids Res.* (2005) 33:D325–8. 10.1093/nar/gki008 15608208 PMC539962

[20] Ruan Z Feng Y. BacWGSTdb, a database for genotyping and source tracking bacterial pathogens. *Nucleic Acids Res.* (2016) 44:D682–7. 10.1093/nar/gkv1004 26433226 PMC4702769

[21] Seeman TKF Page AJ. *Snp-Dists.* San Francisco, CA: GitHub, Inc (2022).

[22] Beres SB Sylva GL Barbian KD Lei B Hoff JS Mammarella ND Genome sequence of a serotype M3 strain of group A *Streptococcus*: phage-encoded toxins, the high-virulence phenotype, and clone emergence. *Proc Natl Acad Sci USA.* (2002) 99:10078–83. 10.1073/pnas.152298499 12122206 PMC126627

[23] Ogura K Watanabe S Kirikae T Miyoshi-Akiyama T. Complete genome sequence and comparative genomics of a *Streptococcus pyogenes* emm3 strain M3-b isolated from a Japanese patient with streptococcal toxic shock syndrome. *J Genomics.* (2017) 5:71–4. 10.7150/jgen.20915 28698738 PMC5504827

[24] Mollick JA Miller GG Musser JM Cook RG Grossman D Rich RR. A novel superantigen isolated from pathogenic strains of *Streptococcus pyogenes* with aminoterminal homology to staphylococcal enterotoxins B and C. *J Clin Invest.* (1993) 92:710–9. 10.1172/JCI116641 8349810 PMC294905

