# Supplementary material for: Invasive Infection With emm3/ST15 Streptococcus pyogenes: The First Case Report From China and Complete Genome Analysis
Source: Front Med (Lausanne). 2022 May 9;9:861087. doi: 10.3389/fmed.2022.861087 (PMC9126071; doi:10.3389/fmed.2022.861087)
Supplement: Supplementary file 2 [file Table_1.DOCX]

**Supplementary Table1.** Antibiotic Susceptibility Test results

| **Antibiotic** | **Disk Content (µg)** | **Zone Diameter (mm)** | **Susceptibility** |
| --- | --- | --- | --- |
| Levofloxacin | 5 | 24 | Susceptible |
| Chloramphenicol | 30 | 28 | Susceptible |
| Clindamycin | 2 | 38 | Susceptible |
| Ceftriaxone | 30 | 28 | Susceptible |
| Erythromycin | 15 | 41 | Susceptible |
| Penicillin | 1 | 30 | Susceptible |
| Tetracycline | 30 | 23 | Susceptible |
| Vancomycin | 30 | 27 | Susceptible |

**Supplementary Table2.** Virulence factors detection in three *emm3*/ST15 GAS strains.

| **Database** | **Gene** | **Product** | **Accession** | **MGAS315 (%Co;%Id)** | **Mb-3**  **(%Co;%Id)** | **SHZ-1**  **(%Co;%Id)** |
| --- | --- | --- | --- | --- | --- | --- |
| vfdb | *cpa* | (cpa) fibronectin-bing protein SfbI | NP_663902 | 100;100 | 100;100 | 100;100 |
| vfdb | *fbaB* | (fbaB) fibronectin-bing protein SfbII | NP_663908 | 100;100 | 82.45;92.58 | 100;100 |
| vfdb | *slo* | (slo) streptolysin O precursor | NP_268546 | 100;99.53 | 100;99.53 | 100;99.53 |
| vfdb | *speg* | (speg) streptococcal exotoxin G precursor | NP_268582 | 100;99.29 | 100;99.29 | 100;99.29 |
| vfdb | *spea* | (spea) streptococcal exotoxin A precursor - phage associated | NP_665105 | 100;100 | 100;100 | 100;100 |
| vfdb | *hylP* | (hylP) hyaluronidase phage associated | NP_268936 | 100;99.51 | 100;99.51 | 100;99.51 |
| vfdb | *spek* | (spek) streptococcal exotoxin SpeK - phage associated | NP_665009 | 100;100 | 100;100 | 100;100 |
| vfdb | *grab* | (grab) protein GRAB (protein G-related alpha 2M-binding protein) | NP_269464 | 68.96;96.67 | 68.96;96.67 | 68.96;96.67 |
| vfdb | *ssa* | (ssa) streptococcal superantigen SSA-phage associated | NP_664724 | 100;100 | 100;100 | ND |
| vfdb | *fbp54* | (fbp54) fibronectin-bing protein FbaA | NP_664456 | 100;100 | 100;100 | 100;100 |
| vfdb | *ideS/mac* | (ideS/mac) immunoglobulin G-degrading enzyme | NP_269065 | 100;99.41 | 100;99.41 | 100;99.41 |
| vfdb | *ska* | (ska) streptokinase A precursor | NP_269944 | 100;96.22 | 100;96.22 | 100;96.22 |
| vfdb | *smeZ* | (smeZ) streptococcal mitogenic exotoxin Z | NP_269959 | 98.01;94.59 | 98.01;94.59 | 98.01;94.59 |
| vfdb | *lmb* | (lmb) laminin-binding surface protein | NP_688239 | 100;99.13 | 100;99.13 | 100;99.13 |
| vfdb | *scpA* | (scpA) C5a peptidase precursor | NP_269970 | 98.22;96.47 | 98.22;96.47 | 98.22;96.47 |
| vfdb | *emm* | (emm) M protein type 3 | NP_665531 | 100;100 | 100;100 | 100;99.94 |
| vfdb | *speB* | (speB) pyrogenic exotoxin B | NP_269985 | 100;99.75 | 100;99.75 | 100;99.75 |
| vfdb | *mf/spd* | (mf/spd) deoxyribonuclease | NP_269989 | 100;98.9 | 100;98.9 | 100;98.9 |
| vfdb | *hasA* | (hasA) hyaluronate synthase | NP_270107 | 100;99.52 | 100;99.52 | 100;99.52 |
| vfdb | *hasB* | (hasB) UDP-glucose 6-dehydrogenase | NP_270108 | 100;99.59 | 100;99.34 | 100;99.5 |
| vfdb | *hasC* | (hasC) UDP-glucose pyrophosphorylase | NP_270109 | 100;99.34 | 100;99.34 | 100;99.34 |

%Co, percentage of coverage; %Id, percentage of identity; ND, not determined.
